# Admission NIHSS score and diabetes as independent predictors of in-hospital early neurological improvement following mechanical thrombectomy: a retrospective cohort study

**DOI:** 10.3389/fneur.2025.1685096

**Published:** 2026-01-19

**Authors:** Chenyang Zhao, Xihua Li, Yi Han, Xuefei Ren, Yaxuan Sun

**Affiliations:** Department of Neurology, Shanxi Provincial People’s Hospital, Shanxi Medical University, Taiyuan, China

**Keywords:** calibration, decision-curve analysis, diabetes mellitus, early neurological improvement, endovascular thrombectomy, ENI-4, NIHSS, prognostic model

## Abstract

**Background:**

Accurate early prognostication in acute ischemic stroke (AIS) is essential for optimizing post-thrombectomy management strategies. However, the predictive utility of baseline clinical characteristics remains underexplored in real-world emergency settings.

**Objective:**

To identify independent clinical predictors of in-hospital neurological improvement following mechanical thrombectomy in AIS patients, with particular focus on admission NIHSS score and comorbid diabetes mellitus.

**Methods:**

In this retrospective single-center cohort study, 250 AIS patients who underwent emergency mechanical thrombectomy between January 2020 and December 2022 were analyzed. Patients were dichotomized according to an in-hospital early neurological improvement endpoint defined *a priori* as ENI-4 (decrease ≥4 points in NIHSS from admission to discharge). All analyses were repeated in sensitivity analyses using two alternative definitions: a clinician-adjudicated composite of in-hospital neurological improvement and discharge NIHSS ≤1/0. Logistic regression analyses were employed to determine independent predictors. Model performance was evaluated using ROC curve analysis, calibration plots, and nomogram construction.

**Results:**

Among the 250 patients, 196 (78.4%) showed neurological improvement during hospitalization. Multivariate logistic regression revealed that a lower admission NIHSS score (OR = 0.867, 95% CI: 0.810–0.927; *p* < 0.001) and absence of diabetes mellitus (OR = 0.357, 95% CI: 0.129–0.988; *p* = 0.047) were independently associated with favorable short-term outcomes. The final model demonstrated moderate discriminative ability (AUC = 0.711) and good calibration. Spline analysis demonstrated a non-linear NIHSS–outcome relationship, and decision-curve analysis showed positive net benefit across 10–30% thresholds. A nomogram based on the model was developed for bedside application. Using ENI-4 as the primary outcome, lower admission NIHSS and absence of diabetes remained independently associated with in-hospital neurological improvement in the multivariable model (NIHSS OR 0.867; diabetes OR 0.357).

**Conclusion:**

Lower NIHSS scores at presentation and non-diabetic status are independent predictors of early neurological improvement following thrombectomy. The internally validated model provides a clinically accessible tool for early risk stratification in AIS patients and may inform post-procedural monitoring and care planning in settings lacking long-term functional follow-up.

## 1 Introduction

Acute ischemic stroke (AIS) continues to represent a major global contributor to neurological disability and cerebrovascular mortality (1, 2). Over the past decade, endovascular thrombectomy has emerged as the standard-of-care for select individuals presenting with large vessel occlusion (LVO), particularly when administered within an appropriate therapeutic time frame, yielding substantial improvements in neurological outcomes (3, 4). Nevertheless, in the context of real-world emergency clinical settings, outcome heterogeneity remains significant—particularly among those with indeterminate neurological profiles at baseline (5–7).

In current neurovascular practice, several pre-intervention parameters—including the National Institutes of Health Stroke Scale (NIHSS) score at presentation, the patient’s functional baseline as estimated by the premorbid modified Rankin Scale (mRS), and the presence of wake-up stroke—are commonly employed to assess candidacy for acute revascularization procedures (8). Although routinely incorporated into triage and risk assessment algorithms, the standalone predictive validity of these variables remains under debate (9–12). On one hand, elevated NIHSS scores may reflect extensive ischemic injury, suggesting unfavorable prognosis (13); on the other hand, some evidence indicates that such patients may harbor salvageable penumbra within occluded proximal vasculature, making them viable candidates for reperfusion therapy with potential for significant recovery (14, 15). Similarly, wake-up stroke—once systematically excluded from major thrombectomy trials—has recently been reconsidered in light of imaging-guided patient selection strategies; however, its prognostic significance within the framework of endovascular intervention remains inconclusive (16, 17). However, despite the widespread availability of simple clinical variables such as admission NIHSS and vascular risk factors, there is still a lack of pragmatic bedside tools that use only these routinely collected measures to predict early in-hospital outcomes after thrombectomy.

To date, few studies have systematically explored how pre-treatment neurological characteristics quantitatively relate to short-term post-procedural recovery, and clinical consensus regarding these predictive markers remains lacking. Addressing this knowledge gap is critical for refining patient selection protocols, enhancing preprocedural communication with patients and caregivers, and informing individualized therapeutic strategies.

The objective of the present investigation is to quantitatively assess the relationship between initial neurological metrics—including NIHSS score upon admission, premorbid mRS status, and stroke onset timing (i.e., wake-up stroke)—and short-term improvement in neurological function following emergency mechanical thrombectomy. To this end, we employed multivariate logistic regression modeling, receiver operating characteristic (ROC) curve analysis, and the construction of a prognostic nomogram to evaluate predictive performance and to generate clinically applicable decision-support tools for early risk stratification.

Unlike most prior studies centered on 90-day mRS or imaging-intensive predictors, we target a pragmatic, in-hospital neurological improvement endpoint that aligns with real-world emergency workflows where long-term follow-up is often infeasible. We focus on a minimal, universally available predictor set—admission NIHSS and diabetes—prioritizing model calibration, internal validation, and bedside deployability via a nomogram. Beyond confirming the directional effects reported previously, we quantify non-linearity, individualized marginal effects, and clinical utility using decision-curve analysis, thereby translating known associations into an actionable early-triage tool.

## 2 Materials and methods

### 2.1 Study design and population

This investigation employed a retrospective cohort design at a single tertiary care facility, Shanxi Provincial People’s Hospital. The study population comprised a consecutive series of individuals diagnosed with acute ischemic stroke (AIS) who received emergency endovascular thrombectomy between January 2020 and December 2022. Eligible cases were identified through the institutional stroke intervention registry and further validated using neuroimaging assessments and electronic health record review. Initially, 254 patients were screened for eligibility.

Enrollment criteria required that participants: (1) were 18 years of age or older; (2) had a radiologically confirmed diagnosis of AIS; (3) underwent mechanical thrombectomy as part of their acute management; and (4) had complete documentation of admission NIHSS score, premorbid functional status as assessed by the modified Rankin Scale (mRS), wake-up stroke classification, and in-hospital outcome measures. Individuals were excluded if critical data points were missing, if they received alternative therapies (such as intravenous thrombolysis alone), or if confounding factors—such as early withdrawal of care or progression to brain death—introduced substantial bias. Additionally, cases with ambiguous discharge status were omitted.

Following the application of these inclusion and exclusion criteria, a total of 250 patients with complete datasets were retained for final analysis. The study protocol received approval from the hospital’s ethics review board, and the requirement for informed consent was formally waived owing to the retrospective nature of the analysis and the anonymization of patient data.

### 2.2 Data collection and variable definitions

Clinical and procedural data were retrospectively retrieved from the hospital’s integrated electronic health information system and the dedicated interventional stroke registry. Baseline variables captured upon admission encompassed patient demographics (e.g., age, sex), established cerebrovascular risk factors (such as hypertension and diabetes mellitus), premorbid functional independence, and the initial neurological deficit severity.

All neuroimaging interpretations, intra-procedural notes, and discharge summaries were independently reviewed by two experienced investigators to ensure reliability and completeness of the dataset.

Primary and secondary outcomes. The primary endpoint was in-hospital early neurological improvement defined as ENI-4, that is, a decrease of ≥4 points in NIHSS score between admission and discharge.

As secondary and sensitivity endpoints, we additionally evaluated (i) a clinician-adjudicated composite of in-hospital neurological improvement versus no improvement, and (ii) discharge NIHSS thresholds (≤1 or = 0). All primary analyses were first performed using ENI-4 and then replicated for these alternative definitions; detailed outcome ascertainment criteria and agreement statistics are provided in the Supplement.

To maximize inter-rater consistency, all discharge assessments were independently conducted by two board-certified neurologists trained in standardized outcome adjudication. Disagreements were adjudicated by a senior consultant neurologist. Although this endpoint does not correspond to the conventional 90-day modified Rankin Scale (mRS), it is a widely recognized and validated surrogate outcome in observational stroke studies where post-discharge follow-up is not feasible (5, 10).

The primary pre-interventional predictor variables assessed in this study included:

Admission NIHSS score, treated as a continuous measure (range: 0–42),

Premorbid mRS score, an ordinal indicator of functional capacity (scale: 0–5),

Wake-up stroke status, a binary classification (yes = 1 if onset occurred during sleep and symptoms were first noticed upon awakening; no = 0 otherwise).

Other covariates included in the multivariate regression models comprised standard demographic parameters and clinical comorbidities, such as patient age, sex, previous history of ischemic stroke, hypertension, and diabetes mellitus. This adjudicated in-hospital endpoint (and ENI-4 / discharge NIHSS ≤1/0 in sensitivity analyses) has been widely used in MT cohorts and real-world stroke registries, facilitating cross-center reproducibility [see (5, 10) and Supplementary methods].

### 2.3 Statistical analysis

All statistical procedures were executed using Python version 3.11, incorporating analytical and visualization libraries such as pandas, statsmodels, scikit-learn, matplotlib, and seaborn. A two-tailed *p*-value of less than 0.05 was considered indicative of statistical significance across all inferential tests.

Continuous data were summarized either as mean ± standard deviation or as median with interquartile range (IQR), depending on their distributional characteristics. Categorical variables were expressed as absolute frequencies and percentages. Between-group comparisons were conducted using independent-samples *t*-tests for normally distributed continuous variables or Mann–Whitney U tests for non-normally distributed data. Chi-square or Fisher’s exact tests were applied to compare proportions, as appropriate.

To identify predictors associated with in-hospital neurological improvement, univariate logistic regression models were first constructed for each candidate variable. Variables yielding *p* values below 0.10 in univariate screening, along with other clinically relevant covariates, were entered into a multivariate logistic regression framework. The final adjusted model included the following predictors: age, sex, NIHSS score on admission, premorbid mRS score, wake-up stroke status, diabetes mellitus, hypertension, and history of prior ischemic stroke. Results were reported as odds ratios (ORs) with corresponding 95% confidence intervals (CIs), and findings were graphically represented using a forest plot (Supplementary Figure S5).

Analytical pathway. First, we fitted a full multivariable logistic regression model including all candidate predictors (age, sex, admission NIHSS, premorbid mRS, wake-up stroke status, hypertension, diabetes mellitus, and history of prior ischemic stroke). Second, we identified variables that remained independently associated with the primary ENI-4 endpoint in this fully adjusted model. Third, to enhance bedside usability, we derived a simplified two-variable clinical model that retained only admission NIHSS and diabetes. All performance metrics (discrimination, calibration, and decision-curve analysis) were computed for both the full and simplified models; the simplified NIHSS + diabetes model is the main focus of the present report, whereas detailed coefficients for the full model are provided in Supplementary Table S4.

Missing data and non-linearity. Missing data were handled using multiple imputation by chained equations (MICE, *m* = 20) under a missing-at-random assumption, pooling estimates with Rubin’s rules. Restricted cubic splines (RCS) were used to evaluate potential non-linear associations between admission NIHSS and the outcome; knot placement followed Harrell’s recommendations with sensitivity to 4–5 knots. We report overall and pointwise Wald tests for non-linearity and visualize partial-dependence/marginal-effect curves.

**Figure 1:**
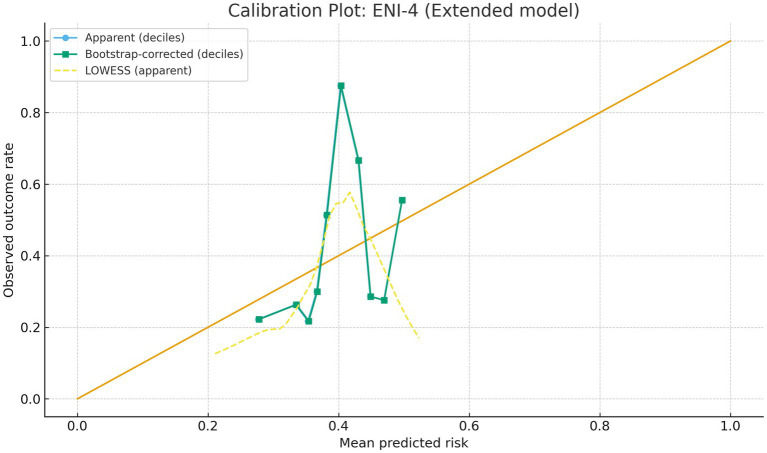
Calibration of the simplified NIHSS + diabetes model for in-hospital ENI-4. The *x*-axis shows deciles of predicted risk and the *y*-axis shows the observed proportion of ENI-4. Apparent and bootstrap-corrected calibration curves are plotted together with the ideal 45° line, and a LOWESS smooth of the apparent relationship is superimposed to illustrate overall agreement between predicted and observed risks. N and event counts, Brier score, and calibration intercept/slope are provided adjacent to the figure or in Supplementary Table S4.

AUCs were computed and are reported in Table 1 and Supplementary Table S4; ROC curves are shown in Supplementary Figure S6. Apparent and bootstrap-corrected calibration with LOWESS smoothing is shown in Figure 1; decile-based summaries are provided in the Supplement. Adjusted odds ratios with 95% confidence intervals are visualized in Supplementary Figure S5, with exact coefficients in Supplementary Table S4.

**Table 1:** Multivariable logistic regression model including all candidate predictors (full model).

Characteristics	OR	95% CI	*P***-**value
Age	0.989	0.963–1.016	0.420
Pre-mRS	1.263	0.747–2.133	0.383
NIHSS on admission	0.867	0.810–0.927	<0.001
Hypertension	0.841	0.427–1.657	0.616
Diabetes	0.357	0.129–0.988	0.047
Stroke	1.161	0.459–2.936	0.753
Gender (male)	1.003	0.503–1.999	0.993
Wake-up stroke (yes)	1.390	0.636–3.038	0.410

Internal validation and calibration. Discrimination was summarized by the area under the ROC curve (AUC) with optimism-correction via 1,000-bootstrap resampling. Calibration was evaluated using calibration-in-the-large (intercept) and calibration slope, with bootstrap-corrected curves presented alongside apparent curves. Brier score and Hosmer–Lemeshow-type smooth calibration (risk deciles) are reported in the Supplement.

Incremental value and clinical utility. To quantify the added value of diabetes beyond NIHSS, we explored changes in discrimination and reclassification between models with and without diabetes [ΔAUC, net reclassification improvement (NRI), and integrated discrimination improvement (IDI)]; detailed estimates for these exploratory metrics are reported in Supplementary material. Decision-curve analysis (DCA) was performed across clinically plausible threshold probabilities (10–30%) with per-100-patients net-benefit interpretation. Model complexity was justified by events-per-variable (EPV); exploratory NIHSS × diabetes interaction and subgroup robustness (e.g., posterior vs. anterior circulation; IVT bridging) were examined. When overfitting signals were present, penalization-based shrinkage (e.g., ridge) was applied, with details in Supplementary methods.

A graphical nomogram was constructed based on the final logistic regression model to estimate individualized probabilities of in-hospital neurological recovery. Internal validation was conducted using bootstrap resampling techniques. The model showed no evidence of overfitting or miscalibration, indicating strong internal validity and potential applicability across similar clinical contexts.

### 2.4 Ethical considerations

This study was conducted in accordance with the principles of the Declaration of Helsinki and institutional ethical guidelines. Given the retrospective design and anonymized data, the requirement for informed consent was waived. The study protocol was approved by the Ethics Committee of Shanxi Provincial People’s Hospital (Approval No.: 609).

### 2.5 Reporting guidelines

This study followed the STROBE (Strengthening the Reporting of Observational Studies in Epidemiology) statement to ensure transparency and completeness of reporting for cohort studies. A completed STROBE checklist is provided in the Appendix.

## 3 Results

### 3.1 Baseline characteristics

The final analytical cohort comprised 250 individuals who underwent emergency mechanical thrombectomy. Using the primary ENI-4 definition, 196 of 250 patients (78.4%) met the criteria for in-hospital early neurological improvement and were classified into the improvement group, whereas 54 patients (21.6%) did not meet the ENI-4 criteria (including those with no improvement, in-hospital mortality, or indeterminate discharge status) and were therefore classified into the non-improvement group. A comparative summary of baseline characteristics between the improvement and non-improvement groups is presented in Table 2.

**Table 2:** Baseline characteristics of patients undergoing emergency mechanical thrombectomy (*N* = 250).

Characteristics	Good outcome	Poor outcome	*P***-**value
	(*N* = 196)	(*N* = 54)	
Age (years)	61.16 ± 13.34	63.20 ± 12.12	0.310
History of diabetes, n (%)	10 (5.1%)	6 (11.1%)	0.216
Gender, n (%)			0.659
Male	132 (67.3%)	34 (63.0%)	
Female	64 (32.7%)	20 (37.0%)	
Wake-up stroke, n (%)			0.302
Yes	56 (28.6%)	11 (20.4%)	
No	140 (71.4%)	43 (79.6%)	
Previous ischemic stroke, n (%)	32 (16.2%)	9 (16.1%)	1.000
Pre-mRS	0.30 ± 0.76	0.26 ± 0.48	0.703
NIHSS on admission	14.02 ± 5.06	17.61 ± 5.37	<0.001
Hypertension, n (%)	68 (34.5%)	22 (39.3%)	0.617

The average age of patients in the improvement group was 61.16 ± 13.34 years, which did not differ significantly from the non-improvement group (63.20 ± 12.12 years; *p* = 0.310). Similarly, gender distribution was comparable: females accounted for 32.7% (*n* = 64) of those with improvement and 37.0% (*n* = 20) among those without (*p* = 0.6591).

The proportion of patients identified as having wake-up stroke was 28.6% in the improvement group versus 20.4% in the non-improvement group, a difference that did not reach statistical significance (*p* = 0.302). Functional status prior to stroke onset, as measured by the premorbid modified Rankin Scale (mRS), showed no substantial difference between groups (0.30 ± 0.76 vs. 0.26 ± 0.48; *p* = 0.7029), indicating similar baseline levels of independence.

Diabetes was more prevalent in the non-improvement group than in the improvement group (11.1% vs. 5.1%, *p* = 0.216), whereas the distributions of hypertension and previous ischemic stroke were similar between groups (39.3% vs. 34.5%, *p* = 0.617, and 16.1% vs. 16.2%, *p* = 1.000, respectively).

In contrast, a significant intergroup difference was observed in initial stroke severity. The mean admission NIHSS score was notably lower in the improvement group (14.02 ± 5.06) compared with the non-improvement group (17.61 ± 5.37), and this difference was statistically robust (*p* < 0.001), highlighting the prognostic relevance of early neurological status.

### 3.2 Univariate analysis

The univariate logistic regression assessment revealed that several baseline clinical parameters exhibited potential predictive relevance regarding functional recovery during hospitalization. Notably, lower admission scores on the National Institutes of Health Stroke Scale (NIHSS) were strongly correlated with improved outcomes (odds ratio [OR] = 0.85, 95% confidence interval [CI]: 0.80–0.91; *p* < 0.001), underscoring the prognostic importance of initial neurological deficit severity. A trend toward significance was observed for the pre-morbid modified Rankin Scale (mRS), with a borderline association suggesting that diminished premorbid functional independence may predispose to poorer recovery trajectories (OR = 0.71, 95% CI: 0.48–1.04; *p* = 0.080).

Although wake-up stroke did not achieve statistical significance in this preliminary model (OR = 1.55, 95% CI: 0.71–3.42; *p* = 0.270), it was retained for further analysis due to its recognized clinical implications in acute stroke presentations. Regarding vascular risk factors, the presence of diabetes mellitus emerged as significantly associated with reduced odds of inpatient neurological improvement (OR = 0.39, 95% CI: 0.15–0.97; *p* = 0.043). Conversely, no significant relationship was detected for hypertension (OR = 0.82; *p* = 0.560) or prior cerebrovascular events (OR = 1.11; *p* = 0.810). Additionally, neither chronological age nor biological sex demonstrated predictive capacity within this unadjusted analytical framework.

### 3.3 Multivariate analysis

In the primary ENI-4–based multivariable logistic regression model including all candidate predictors (age, sex, admission NIHSS, premorbid mRS, wake-up stroke, hypertension, diabetes, and previous ischemic stroke), only admission NIHSS and diabetes remained independently associated with in-hospital early neurological improvement. Lower NIHSS at presentation was associated with higher odds of ENI-4 (OR 0.87, 95% CI 0.81–0.93; *p* < 0.001), underscoring the prognostic importance of initial stroke severity in early recovery. In contrast, diabetes mellitus was an independent adverse predictor (OR 0.36, 95% CI 0.13–0.99; *p* = 0.047), indicating a lower likelihood of early improvement among patients with pre-existing diabetes.

Other variables, including wake-up stroke status (OR 1.39; *p* = 0.410), premorbid mRS (OR 1.26; *p* = 0.380), and hypertension (OR 0.84; *p* = 0.620), did not show statistically significant associations with ENI-4. These adjusted effect estimates are summarized in the forest plot (Supplementary Figure S5), which highlights that only admission NIHSS and diabetes remained significant in the final regression model. Based on these findings, we derived a parsimonious two-variable clinical model including only admission NIHSS and diabetes; this simplified model underlies the nomogram and performance analyses reported below.

### 3.4 Model performance and visualization

The base model including admission NIHSS alone achieved an AUC of 0.69, whereas the extended simplified model including NIHSS plus diabetes achieved an AUC of 0.71. For the simplified NIHSS + diabetes model, the AUC of 0.71 indicated acceptable discrimination.

**Figure 2:**
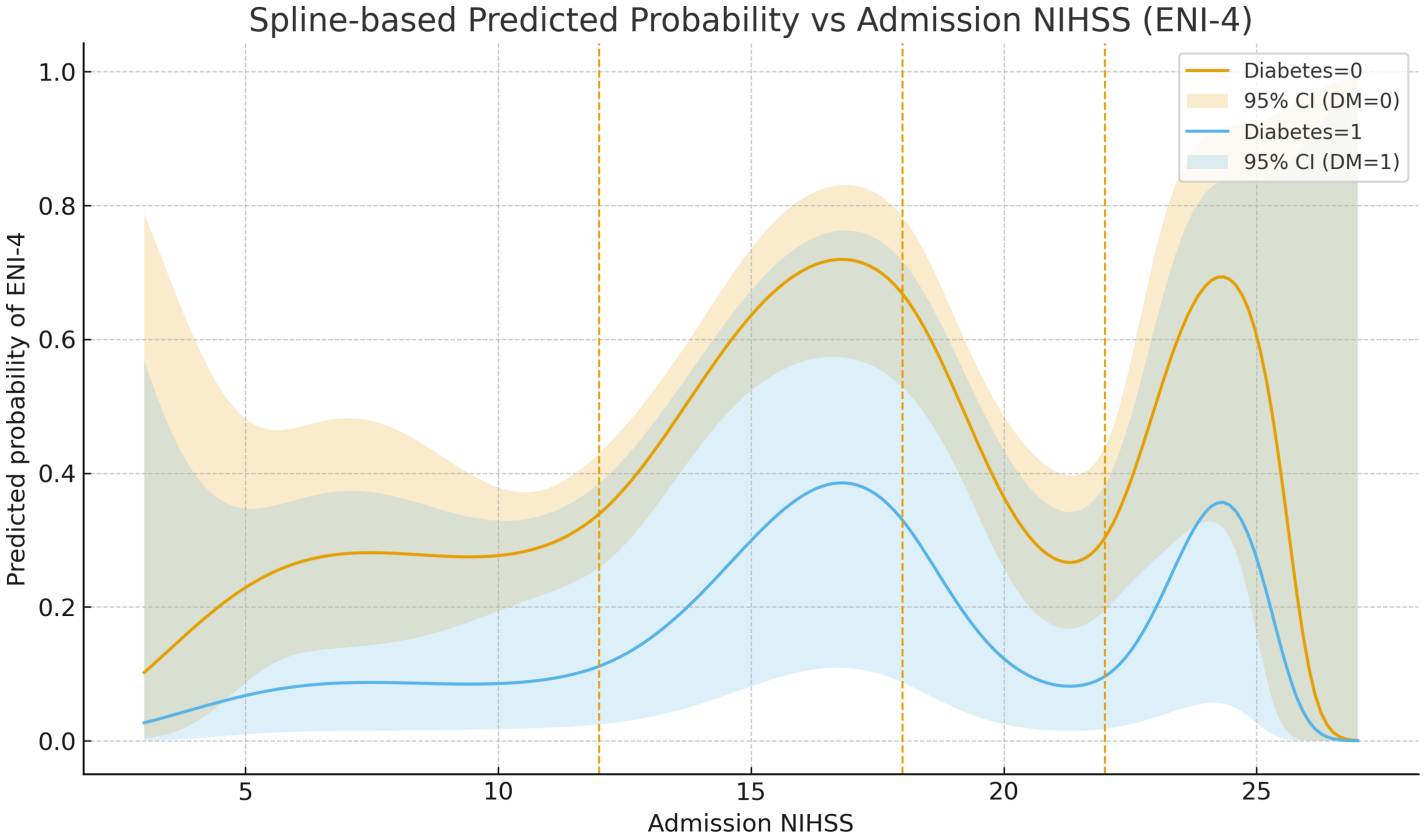
Restricted cubic spline showing the relationship between admission NIHSS and the predicted probability of in-hospital ENI-4 in the simplified NIHSS + diabetes model. The *x*-axis shows admission NIHSS and the *y*-axis shows the predicted probability of ENI-4. The solid line represents the point estimate and the shaded area the 95% confidence band; interior knot locations are indicated on the *x*-axis.

Restricted cubic splines demonstrated a non-linear association between admission NIHSS and ENI-4, with a steeper probability gradient at intermediate NIHSS values (approximately the lower-to-mid-teens) and relative flattening at the extremes (Figure 2) (likelihood-ratio test for non-linearity *p* = 0.0026). Across the entire NIHSS range, the predicted probability curve for patients with diabetes was shifted downward compared with those without diabetes. Marginal-effect curves showed that reductions in NIHSS within the intermediate range were associated with clinically meaningful absolute increases in the probability of in-hospital improvement, whereas comparable changes at very low or very high NIHSS had smaller effects. Full partial-dependence plots are provided in Supplementary Figure S2.

**Figure 3:**
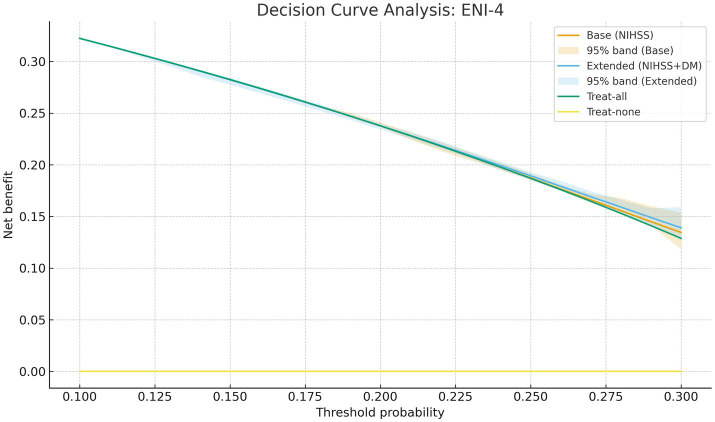
Decision-curve analysis for in-hospital ENI-4 comparing the base model (admission NIHSS alone) and the simplified NIHSS + diabetes model. The *x*-axis shows the threshold probability and the *y*-axis shows the net benefit. Solid lines represent the net benefit of each model with bootstrap-derived 95% confidence bands, while the “treat-all” and “treat-none” strategies are displayed for comparison.

Decision-curve analysis showed that the NIHSS + diabetes model yielded positive net benefit across threshold probabilities of 10–30%, outperforming treat-all and treat-none strategies (Figure 3). At a 20% threshold, the extended model identified more patients who experienced ENI-4 while avoiding unnecessary escalation of care in patients unlikely to improve, suggesting practical utility for ward-level planning. Threshold-specific net-benefit values and corresponding per-100-patient interpretations are provided in Supplementary Table S3.

Calibration of the extended model was acceptable on both apparent and bootstrap-corrected curves with LOWESS smoothing (Figure 1); full decile-based calibration tables are provided in the Supplement. Visual inspection of calibration plots indicated good agreement between predicted and observed risks across risk strata, and decision-curve analysis suggested a consistent net clinical benefit over a wide range of clinically relevant threshold probabilities.

## 4 Discussion

This retrospective cohort study, conducted at a single tertiary stroke center, analyzed 250 patients with acute ischemic stroke (AIS) who underwent emergency mechanical thrombectomy. Our results identified lower admission NIHSS and absence of diabetes mellitus as independent predictors of in-hospital early neurological improvement (ENI-4). These variables were integrated into a multivariable logistic regression model, from which an interpretable bedside risk estimate can be directly derived; full coefficients and CIs are provided in Supplementary Table S4. The model showed moderate discriminative ability (AUC = 0.711) with robust calibration (Figure 1). Non-linear NIHSS effects (Figure 2) and positive decision utility across clinically relevant thresholds (Figure 3) support early triage and ward-level planning. Beyond corroborating these directions, our contribution is to translate them into an internally validated, well-calibrated, and decision-useful model that supports real-time ward-level planning.

Comparison with prior evidence. Prior work has primarily emphasized 90-day mRS and imaging-dependent predictors or early-timepoint NIHSS changes. In contrast, our study focuses on an in-hospital, adjudicated endpoint tailored to emergency workflows where standardized long-term follow-up is not guaranteed, and it deliberately confines predictors to admission NIHSS and diabetes to maximize universality and bedside deployability. By demonstrating good calibration, bootstrap-corrected discrimination, non-linear NIHSS effects, and decision-utility across clinically relevant thresholds, we extend the literature from directional associations to a calibrated, minimal-predictor tool that is actionable for early triage and ward-level planning. Unlike prior meta-analyses focused on long-term mRS or imaging-heavy predictors, our study quantifies non-linearity and clinical utility for a pragmatic in-hospital endpoint, offering a deployable tool when 90-day outcomes are unavailable.

These findings align with prior research underscoring the prognostic utility of initial neurological impairment and vascular risk burden in AIS. For instance, studies by Meyer et al. and Kniep et al. affirmed that early NIHSS scores—particularly at 24 h or discharge—serve as reliable surrogates for long-term functional recovery, particularly when mRS data are incomplete or delayed (5, 10). Likewise, the negative prognostic influence of diabetes mellitus has been well documented, potentially attributable to compromised collateral perfusion, exacerbated oxidative stress, and an increased propensity for hemorrhagic transformation in hyperglycemic states (18–22).

Although wake-up stroke (WUS) has garnered increasing clinical attention in the wake of advanced neuroimaging protocols and extended time-window trials (23, 24), it did not emerge as a statistically significant predictor in either univariate or multivariate analyses in our cohort. This may reflect the neutralizing effect of rapid reperfusion therapy and individualized patient selection, suggesting that WUS per se does not inevitably portend poorer short-term outcomes when treated appropriately.

Clinically, the nomogram we constructed provides an accessible bedside tool that visually quantifies the relative impact of key pre-treatment variables on short-term neurological improvement. This facilitates real-time clinical decision-making, particularly in emergency care environments where standardized 90-day outcome data are not immediately available.

Clinical implications. In settings lacking standardized 90-day outcomes, an in-hospital endpoint supports early rehabilitation referral, monitoring intensity, and discharge coordination. The marginal-effect profiles indicate that moderate reductions in NIHSS within the mid-range translate into meaningful absolute gains in improvement probability, while the absence of diabetes confers additional benefit at the same NIHSS level. Coupled with positive net benefit on DCA, these findings justify integrating the nomogram into bed-planning, care-level escalation/de-escalation, and family counseling during acute hospitalization.

For example, according to our nomogram, a non-diabetic patient presenting with a moderate stroke severity (e.g., NIHSS 12) has a substantially higher predicted probability of in-hospital ENI-4 than a diabetic patient presenting with more severe deficits (eg, NIHSS 18). In practice, such differences may inform the intensity of neurological monitoring, the timing of early rehabilitation referral, and how clinicians counsel patients and families regarding expected in-hospital recovery.

Several strengths underpin this study: consecutive real-world enrollment, a prespecified in-hospital endpoint with blinded dual-adjudication and senior arbitration, and rigorous internal validation with optimism-corrected discrimination and calibration. These features enhance reproducibility while keeping the predictor set minimal and universally available.

First, the retrospective, single-center design inherently restricts the external generalizability of our findings. Second, in the absence of standardized 90-day mRS outcomes, we adopted in-hospital neurological improvement as a surrogate endpoint. While this approach has precedent and has been validated in real-world stroke cohorts (16, 25–27), it may underestimate delayed functional recovery trajectories. Third, key neuroimaging biomarkers—such as ASPECTS scores or perfusion imaging parameters—were not incorporated into the final model, potentially limiting predictive granularity (28). Statistical adequacy was supported by a favorable events-per-variable (EPV) ratio for the final model, and bootstrap-corrected estimates indicated stable discrimination together with calibration-in-the-large and a slope close to ideal. Given the single-center retrospective design, external validation is required to assess geographic and temporal transportability, and we have prespecified a multicenter validation plan to evaluate the performance of both the in-hospital endpoint and the model parameters.

Future research should aim to externally validate this model in multicenter populations, assess its applicability in integrated stroke care pathways, and explore the additive value of incorporating advanced imaging data, serum biomarkers, or machine learning–driven prediction algorithms (29–31). In an era of rapidly evolving AIS management, there remains an urgent need for interpretable, scalable, and clinically deployable tools that support early outcome prediction and guide personalized intervention strategies.

## 5 Conclusion

Lower admission NIHSS scores and absence of diabetes mellitus independently predict in-hospital early neurological improvement (ENI-4) after mechanical thrombectomy. The prognostic model derived from these variables, presented as a clinically interpretable nomogram, demonstrates moderate discriminatory power and favorable calibration performance. This tool offers a rapid, bedside-accessible means of estimating short-term neurological outcomes in acute care environments where long-term follow-up data are unavailable. Despite inherent limitations of the retrospective design, the model may support early risk stratification, facilitate individualized treatment planning, and help identify patients requiring closer surveillance or adjunctive interventions during the acute hospitalization phase. Rather than re-stating known directions of effect, we translate them into a calibrated, minimal-predictor tool with demonstrated decision utility for real-time ward-level planning when 90-day outcomes are unavailable.

## Data Availability

De-identified data supporting the conclusions of this article will be made available by the authors upon reasonable request, in accordance with institutional policies and applicable regulations. The analysis code will also be made available upon reasonable request.
