# Supplementary material for: Admission NIHSS score and diabetes as independent predictors of in-hospital early neurological improvement following mechanical thrombectomy: a retrospective cohort study
Source: Front Neurol. 2026 Jan 19;16:1685096. doi: 10.3389/fneur.2025.1685096 (PMC12862942; doi:10.3389/fneur.2025.1685096)
Supplement: Supplementary file 1 [file Table_1.docx]

| **Characteristics** | **Good outcome** | **Poor outcome** | ***P***-value |
| --- | --- | --- | --- |
|  | **(N=196)** | **(N=54)** |  |
| **Age(years)** | 61.16 ± 13.34 | 63.20 ± 12.12 | 0.3102 |
| **Diabetes** | 0.05 ± 0.22 | 0.12 ± 0.43 | 0.2163 |
| **Gender,n(%)** |  |  | 0.6591 |
| **Male** | 132 (67.3%) | 34 (63.0%) |  |
| **Female** | 64 (32.7%) | 20 (37.0%) |  |
| **Wake-up stroke,n(%)** |  |  | 0.3024 |
| **Yes** | 56 (28.6%) | 11 (20.4%) |  |
| **No** | 140 (71.4%) | 43 (79.6%) |  |
| **Stroke,n(%)** | 32 (16.2%) | 9 (16.1%) | 1.0000 |
| **Pre-mRS** | 0.30 ± 0.76 | 0.26 ± 0.48 | 0.7029 |
| **NIHSS on admission** | 14.02 ± 5.06 | 17.61 ± 5.37 | ＜0.0001 |
| **Hypertension** | 68 (34.5%) | 22 (39.3%) | 0.6174 |
