# Supplementary material for: Admission NIHSS score and diabetes as independent predictors of in-hospital early neurological improvement following mechanical thrombectomy: a retrospective cohort study
Source: Front Neurol. 2026 Jan 19;16:1685096. doi: 10.3389/fneur.2025.1685096 (PMC12862942; doi:10.3389/fneur.2025.1685096)
Supplement: Supplementary file 2 [file Table_2.docx]

| **Characteristics** | **OR** | **95% CI (lower limit – upper limit)** | ***P***-value |
| --- | --- | --- | --- |
| **Age** | 0.989 | 0.963 – 1.016 | 0.420 |
| **Pre-mRS** | 1.263 | 0.747 – 2.133 | 0.383 |
| **NIHSS on admission** | 0.867 | 0.810 – 0.927 | ＜0.001 |
| **Hypertension** | 0.841 | 0.427 – 1.657 | 0.616 |
| **Diabetes** | 0.357 | 0.129 – 0.988 | 0.047 |
| **Stroke** | 1.161 | 0.459 – 2.936 | 0.753 |
| **Gender (male)** | 1.003 | 0.503 – 1.999 | 0.993 |
| **Wake-up stroke(Yes)** | 1.390 | 0.636 – 3.038 | 0.410 |
