# Supplementary figures and images for: Admission NIHSS score and diabetes as independent predictors of in-hospital early neurological improvement following mechanical thrombectomy: a retrospective cohort study
Source: Front Neurol. 2026 Jan 19;16:1685096. doi: 10.3389/fneur.2025.1685096 (PMC12862942; doi:10.3389/fneur.2025.1685096)

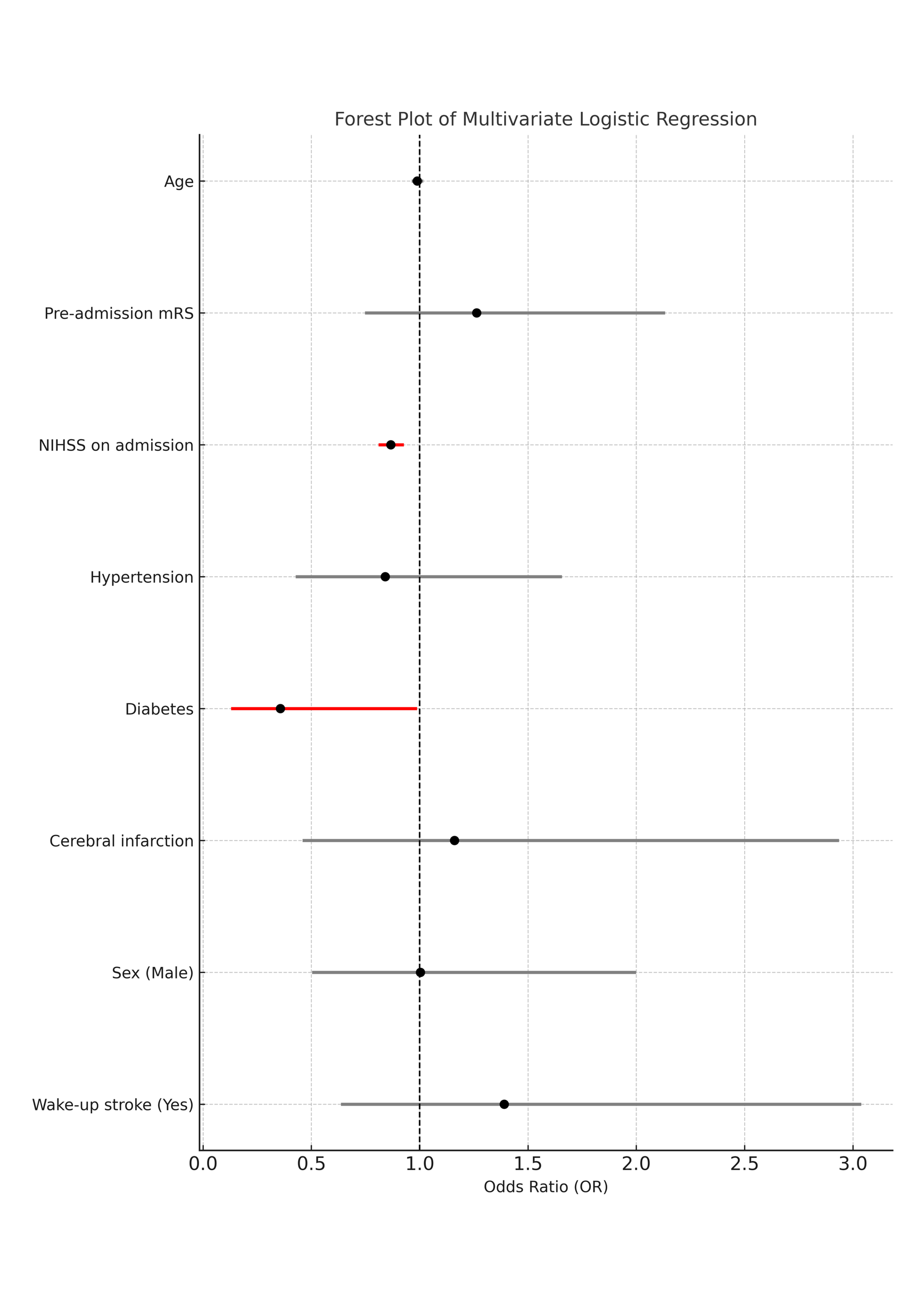

Supplement: Supplementary file 3 [file Image_1.tif]

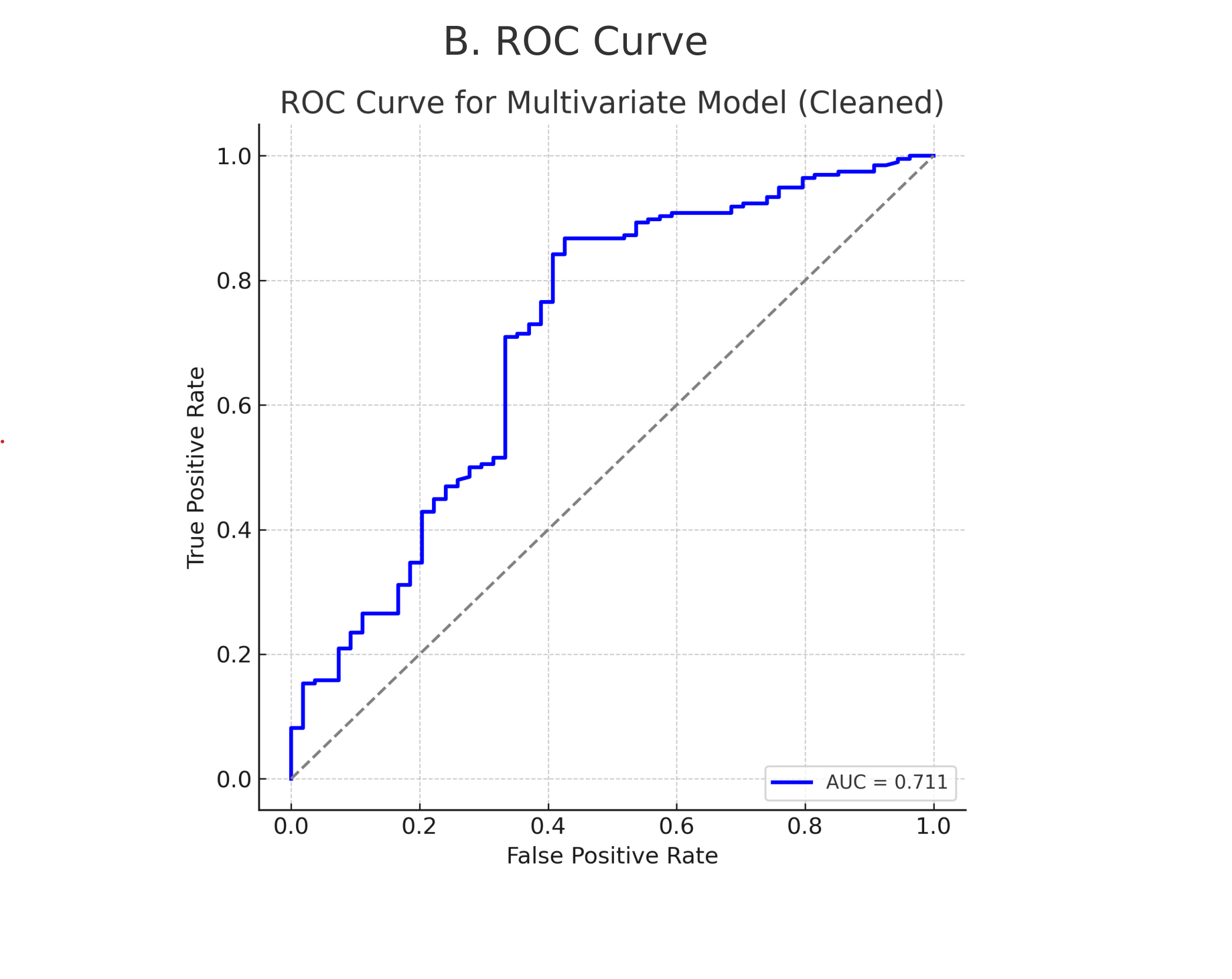

Supplement: Supplementary file 4 [file Image_2.tiff]
